# Catheter-related bloodstream infections in infants hospitalized in neonatal intensive care units: a single center study

**DOI:** 10.1038/s41598-022-17820-w

**Published:** 2022-08-11

**Authors:** Julian F. Kochanowicz, Agnieszka Nowicka, Salwan R. Al-Saad, Lukasz M. Karbowski, Janusz Gadzinowski, Dawid Szpecht

**Affiliations:** 1 Poznan University of Medical Sciences, Poznan, Poland; 2 Chair and Department of Neonatology, Poznan University of Medical Sciences, Poznan, Poland

**Keywords:** Infection, Predictive markers, Bacterial infection, Neonatal sepsis, Neonatal sepsis, Neonatology, Preterm birth, Outcomes research, Risk factors

## Abstract

Sepsis in neonates carries a high morbidity and mortality rate and is among the most feared complications in the neonatal intensive care unit (NICU). Catheter-related bloodstream infections (CRBSI) are a common etiology of late-onset sepsis. The aim of this study was to compare risk factors and characteristics between patients according to the type of catheter that was utilized and according to birth weight classification. The study included 51 newborns with confirmed CRBSI, which were hospitalized in our level 3 NICU between January 2017 and December 2018. The study population was stratified according to the type of venous catheter utilized (peripherally inserted central catheter, central venous catheter (CVC), and peripheral venous catheter). Infants with low birth weight and those who required prolonged parenteral nutrition were most likely to develop CRBSI in our study group. The type of venous catheter was not associated with blood culture results. Also, infants with a birth weight of < 1500 g and > 1500 g did not differ in sepsis etiology. Further research is required to assess venous catheters relative risk of causing sepsis and if the outcome can be traced back specifically to catheter type or patient characteristics.

## Introduction

Venous catheters are among the most commonly used medical tools in practice and are an essential component in the treatment and care of patients in intensive care units. In neonates, they are primarily used to administer medications, obtain blood tests as well as provide parenteral nutrition to vulnerable premature infants^1^. A frequently feared complication of venous catheters is catheter-related bloodstream infections (CRBSI). CRBSI is among the most common causes of late-onset sepsis (LOS) and is described as the development of bacteremia due to catheter placement intravenously^2^. Infants who develop CRBSI have an estimated mortality rate of 70% and may experience impairments in neurodevelopment and growth later in life^3^. Sepsis in neonates is variably classified as late-onset when signs appear at or following the third day until the seventh day of life^4,5^. Most commonly early-onset sepsis is defined as sepsis diagnosed before 72 h of life^6^. Such a classification helps the physician to make the best decision when it comes to starting an empirical antibiotic therapy due to the varying etiologies between these two types of sepsis. A better understanding of this multifactorial complication and its associated risk factors may help to initiate life-saving treatment earlier and to prevent its progression into further stages.

Advancements in modern neonatology have led to greater survivability of infants that have been born preterm, which in turn increased the population that is at risk for LOS due to the increased need for invasive monitoring and support in this population^7^. However, such progress might additionally expose neonates to the dangers of infection. CRBSI can be caused by either the patient's skin flora that gains access to the bloodstream or hospital-derived organism which could lead to nosocomial bacteremia^2^. Increasing signs of sepsis in patients with venous catheters, in the absence of other sources of infection, allows the physician to presume the source of the infection to be catheter-related^8^.

The aim of this study was to compare risk factors as well as characteristics of neonates with CRBSI according to the type of venous catheter that was utilized and according to birth weight classification. Based on these findings we made conclusions about the pathogenesis of sepsis in our studied population, and how to optimize the treatment approach for patients with this complication.

## Materials and methods

### Inclusion criteria

The study included infants with diagnosed septicemia at or after the 7th day of life (LOS) and had confirmed CRBSI. Each participant was allowed to have only one venous catheter at the time of diagnosis: a peripherally inserted central catheter ((PICC) VYGON GmbH & Co.KG), a central venous catheter ((CVC) BD Neoflon), or a peripheral venous catheter ((PVC) Arrow Medical). Infants that developed signs of sepsis before the 7th day of life or that developed septicemia due to factors other than catheter placement were excluded. To exclude urinary tract infections or pneumonia as the source of the infection, we only included patients with negative urine and bronchoscopic cultures.

### Study population

This cross-sectional study included 51 newborns, which were hospitalized between January 2017, and December 2018, at the Neonatal Intensive Care Unit of the Obstetrics & Gynecology Clinical Hospital of the Poznań University of Medical Sciences. During this time a total of 985 newborns were admitted to the level 3 NICU, and all of them were on a ventilator. Data from secondary sources was used for the study, which included the patient’s medical records from the moment of admission until discharge from the clinic. Informed consent about participation in the study has been obtained by the parent and/or legal guardian of all subjects involved in the study.

### Diagnosis

Sepsis was diagnosed based on the presence of two clinical signs and two positive laboratory tests. Clinical signs of sepsis are reluctance to feed, gastrointestinal retention, flatulence, hyperbilirubinemia, loss of muscle tone, bradycardia or tachycardia, tachypnea or apnea, and hypothermia or hyperthermia without disturbance of peripheral perfusion. Laboratory signs of sepsis are increased serum procalcitonin, increased serum CRP, and leukocytosis.

CRBSI was diagnosed when the same organisms grew from a percutaneous blood culture and from a culture from the tip of the venous catheter.

Performing a blood culture was recognized as the gold standard in the diagnosis of sepsis and is considered a compulsory test on suspicion. Blood samples of 0.5–1 ml were drawn from a freshly placed venous access site to subsequently be analyzed with the bacT/ALERT 3D microbial identification system. The blood was cultured using the automatic incubation system as recommended by the manufacturer. Strict aseptic measures were taken to exclude any false contamination during blood sampling, i.e. with surgical washing of hands, wearing a sterile gown, and sterile gloves. The collection site is protected by surgical covers. The skin at the injection site was disinfected with antiseptic alcohol by making circular movements in a centrifugal direction. After aspirating the blood with a sterile syringe, the puncture site was disinfected, and covered with a sterile dressing. The blood was taken during a period at which the probability to isolate a pathogenic organism is the greatest. Generally, the blood sample is taken before antibiotic therapy is initiated, however, in certain cases where this approach was not possible, the sample was taken before the next antibiotic was given.

### Methods

The studied group was stratified based on two criteria. The first one is the type of venous catheter. Body weight was used as the second criterion and we differentiated between infants with a body weight of less than or more than 1500 g. Within each subpopulation, we assessed the child’s birth history and made divisions according to gender, birth weight, gestational age, type of pregnancy, mode of delivery, place of birth, and APGAR score at the first, third, and fifth minute of life. Of interest was also the day of life that the venous catheter was inserted, the duration of its use, type of antibiotic therapy, day of parenteral nutrition termination, day of life at which sepsis occurred, and hospitalization time. Laboratory parameters that were taken into account included C-reactive protein, procalcitonin, white blood cell count, and blood culture.

### Statistical analysis

The statistical analysis of the neonatal population was performed in two stages. In the first stage, the frequency by which certain parameters occurred was described with the use of descriptive analysis. Secondly, the correlation between certain variables was analyzed. Dispersion of data in relation to the center was represented using mean (standard deviation) in continuous variables. For categorical variables, median (interquartile range (IQR)) was utilized. Variables represented on a nominal scale were presented using modified number tables.

The Shapiro–Wilk test was used to test for compliance and data normality. To assess the correlations between certain variables, nonparametric tests such as Mann–Whitney test, Kruskal-Willis test, Dann post-hoc test, and the independent chi-square test were used. The level of significance was set at α = 0.05. Tables were used to present the collected data. All statistical calculations were performed with the help of R Statistics Version 4^9^ and IBM SPSS Statistics Version 26.0^10^.

### Institutional review board statement

The study was conducted according to the guidelines of the Declaration of Helsinki, and approved by the Institutional Review Board of Department of Neonatology of the Poznan University of Medical Sciences.

### Informed consent statement

Informed consent about the participation in the study has been obtained by the parent and/or legal guardian of all subjects involved in the study.

## Results

The study included 29 males and 22 females, which had a mean birth weight of 1636.86 ± 896.17 g and a mean gestational age of 30.71 ± 4.37 weeks. The median and interquartile range of APGAR scores at the first, third, and fifth minute of life was 6 (4–6), 7 (5–8), and 8 (6.25–9), respectively. Thirty-eight children (74.51%) were born by cesarean section. Out of the 51 infants included in the study, the majority received a PICC (22/51–43.1%) or a CVC (16/51–31.4%), while 13/51 (25.5%) infants had a PVC.

A significant difference was observed in gestational age (*p* = 0.001) and birth weight (*p* ≤ 0.001) between the infants. On average, preterm infants with higher gestational age (33.50 ± 4.59 weeks) were receiving a CVC.

Subjects with a PICC and those with a PVC were born at 31.38 ± 3.66 weeks and 28.41 ± 3.32 weeks of gestation, respectively (*p* = 0.001). The newborns that received a CVC had the highest birth weight at a mean of 2344.06 ± 1030.43 g followed by the patients with a PVC, which, on average, weighed 1590.0 ± 548.04 g. Patients with a PICC had the lowest birth weight at 1150.23 ± 616.90 g. The catheter was inserted at the earliest for PVC infants (1.69 ± 1.55 days of age) followed by PICC infants (4.95 ± 3.79 days of age), and CVC infants (10.06 ± 17.10 days of age) (*p* = 0.001). Termination of parenteral nutrition was latest for patients with a CVC (31.63 ± 20.64 days), while for patients with a PICC termination occurred at 29.0 ± 15.84 days of age. PVC patients were fed parenterally until they were 17.54 ± 8.03 days old (*p* = 0.031) and required the shortest course of antibiotic therapy (10.69 ± 2.18 days). Babies with a PICC and a CVC were treated for 12.64 ± 2.82 and 18.13 ± 9.05 days, respectively (*p* = 0.001). On average PICC patients were hospitalized the longest (86.36 ± 37.28 days), followed by CVC patients at a mean of 51.0 ± 27.76 days and PVC patients at 48.23 ± 28.72 days (*p* = 0.002).

C-reactive protein was the only laboratory value that was significantly associated with the type of catheter used. The highest mean CRP was measured in the patients with the highest gestational age (those that received a CVC) (74.57 ± 40.47 µg/mL), while patients with a PVC had 59.31 ± 39.93 µg/mL, and PICC patients had 48.48 ± 62.52 µg/mL (*p* = 0.015).

Staphylococcus epidermidis was the most commonly isolated pathogen from the blood of our subjects (16/51–31.37%) followed by staphylococcus haemolyticus that infected 15 (29.41%) subjects. Other bacteria isolated from the subject’s blood included enterococcus faecalis (6/51–11.76%), staphylococcus capitis (5/51–9.80%), staphylococcus aureus (3/51–5.88%), klebsiella pneumoniae (3/51–5.88%), escherichia coli (2/51–3.92%), staphylococcus hominis (2/51–3.92%), serratia marcescens (2/51–3.92%), staphylococcus saprophyticus (1/51–1.96%), staphylococcus warneri (1/51–1.96%), staphylococcus caprae (1/51–1.96%), klebsiella oxytoca (1/51–1.96%), and bacillus sporothermodurans (1/51–1.96%). The results from blood culture were not significantly correlated with the type of central line that has been used (*p* = 0.361).

All findings for the studied population stratified by type of venous catheter can be found in Table 1.

**Table 1:** Characteristics of the assessed population based on the type of venous catheter used.

	PICC (n = 22)	CVC (n = 16)	PVC (n = 13)	*p*-value
**Gender**				0.552
Male	13 (59.09%)	10 (62.50%)	6 (46.15%)	
Female	9 (40.91%)	6 (37.50%)	7 (53.85%)	
**Gestational age**	31.38 ± 3.66	33.50 ± 4.59	28.41 ± 3.32	0.001
Mean ± (SD) (weeks)				
**Birth weight**	1150.23 ± 616.90	2344.06 ± 1030.43	1590.00 ± 548.04	< 0.001
Mean ± (SD) (grams)				
**Mode of delivery**				0.566
Caesarean section	18 (81.82%)	12 (75.00%)	8 (61.54%)	
Vaginal	4 (18.18%)	4 (25.00%)	5 (38.46%)	
**APGAR 1 min**	4.5 (3.75–7)	8 (4–9.75)	6 (5–8.5)	0.543
Median (IQR)				
**APGAR 3 min**	6 (5–7.75)	7 (6–9)	6 (5–8)	0.420
Median (IQR)				
**APGAR 5 min**	7 (6–8)	8 (7–10)	8 (6.25–9)	0.187
Median (IQR)				
**Age at venous catheter insertion**	4.95 ± 3.79	10.06 ± 17.10	1.69 ± 1.55	0.001
Mean ± (SD) (days)				
**Duration of venous catheter use**	23.82 ± 15.45	27.06 ± 19.99	21.38 ± 3.71	0.582
Mean ± (SD) (days)				
**Age at diagnosis of sepsis**	25.77 ± 26.24	24.06 ± 22.27	14.38 ± 12.73	0.103
Mean ± (SD) (days)				
**Age at termination of parenteral nutrition**	29.00 ± 15.84	31.63 ± 20.64	17.54 ± 8.03	0.031
Mean ± (SD) (days)				
**Duration of antibiotic therapy**	12.64 ± 2.82	18.13 ± 9.05	10.69 ± 2.18	0.001
Mean ± (SD) (days)				
**Duration of hospitalization**	86.36 ± 37.28	51.00 ± 27.76	48.23 ± 28.72	0.002
Mean ± (SD) (days)				
**Laboratory results**				
**C-Reactive protein (CRP)**	48.48 ± 62.52	74.57 ± 40.47	59.31 ± 39.93	0.015
Mean ± (SD) (mg/L)				
**Procalcitonin (PCT)**	26.30 ± 26.54	23.75 ± 29.35	20.22 ± 19.53	0.395
Mean ± (SD) (ng/mL)				
**White blood cells (WBCs)**	30.14 ± 16.02	25.10 ± 13.91	19.56 ± 4.71	0.074
Mean ± (SD) (×10^9^/L)				
**Blood culture results**				
*Staphylococcus haemolyticus*	4 (18.18%)	6 (37.50%)	5 (38.46%)	0.361
*Staphylococcus epidermidis*	8 (36.36%)	3 (18.75%)	5 (38.46%)	
Others*	10 (45.46%)	7 (43.75%)	3 (23.08%)	

PICC, Peripherally Inserted Central Catheter; CVC, Central Venous Catheter; PVC, Peripheral Venous Catheter; CRP, C-reactive protein; PCT, procalcitonin. *Other bacteria include: *enterococcus faecalis, staphylococcus capitis, staphylococcus aureus, klebsiella pneumoniae, escherichia coli, staphylococcus hominis, serratia marcescens, staphylococcus saprophyticus, staphylococcus warneri, staphylococcus caprae, klebsiella oxytoca, and bacillus sporothermodurans.*

Furthermore, we stratified the study population into two weight groups. The population consisted of 27/51 (52.94%) infants that weighed less than 1500 g, while 24/51 (47.06%) weighed more than 1500 g.

Infants with lower birth weight had a lower gestational age (27.77 ± 2.21 weeks (*p* = 0.001)), lower APGAR score at the first (5 (4–6) (*p* = 0.006)), and fifth minute (7 (6–8) (*p* = 0.010)) of life. Hospitalization time was comparatively longer in lower birth weight infants (79.96 ± 34.54 days (*p* = 0.003)). The most common catheter used in these newborns were PICCs (19/27 (70.38%)), whereas CVCs and PVCs were each used in 4/27 (14.81%) (*p* = 0.001).

Infants with a higher birth weight had a higher APGAR score at the first (7.5 (5–9) (*p* = 0.006)) and fifth minute (8 (7–10) (*p* = 0.010)) of life. Hospitalization time was comparatively shorter in the higher birth population (51.29 ± 33.91 days (*p* = 0.003)). The most common catheter used in these newborns was a CVC (12/24 (50.00%)), whereas 9/24 (37.50%) used PVCs and 3/24 (12.5%) used PICCs (*p* = 0.001). The results from blood culture were not significantly correlated with the birth weight classification (*p* = 0.262).

All findings of the studied population stratified by body weight can be found in Table 2.

**Table 2:** Characteristics of assessed weight groups based on greater than or less than 1500 g in weight.

	< 1500 g (n = 27)	> 1500 g (n = 24)	*p*-value
**APGAR 1 min**	5 (4–6)	7.5 (5–9)	0.006
Median (IQR)			
**APGAR 3 min**	6 (5–7)	7.5 (6–9)	0.069
Median (IQR)			
**APGAR 5 min**	7 (6–8)	8 (7–10)	0.010
Median (IQR)			
**Age at venous catheter insertion**	4.54 ± 3.34	6.97 ± 6.53	0.401
Mean ± (SD) (days)			
**Duration of antibiotic therapy**	12.61 ± 3.35	15.13 ± 8.19	0.478
Mean ± (SD) (days)			
**Duration of hospitalization**	79.96 ± 34.54	51.29 ± 33.91	0.003
Mean ± (SD) (days)			
**Venous catheter type**			0.001
PICC	19 (70.38%)	3 (12.50%)	
CVC	4 (14.81%)	12 (50.00%)	
PVC	4 (14.81%)	9 (37.50%)	
**Age at termination of parenteral nutrition**	27.71 ± 11.95	26.33 ± 21.08	0.149
Mean ± (SD) (days)			
**Laboratory results**			
**C-Reactive protein (CRP)**	61.56 ± 60.47	57.02 ± 39.97	0.811
Mean ± (SD) (mg/L)			
**Procalcitonin (PCT)**	25.03 ± 24.67	22.74 ± 26.95	0.340
Mean ± (SD) (ng/mL)			
**White blood cells (WBCs)**	27.23 ± 13.00	24.32 ± 14.72	0.142
Mean ± (SD) (×10^9^/L)			
**Blood culture results**			
*Staphylococcus haemolyticus*	7 (25.00%)	8 (33.33%)	0.262
*Staphylococcus epidermidis*	7 (25.00%)	9 (37.50%)	
*Others	14 (50.00%)	7 (29.17%)	

## Discussion

Neonatal sepsis is one of the most feared complications in the NICU due to its high morbidity and mortality rate and possible neurological and pulmonological developmental impairments^11,12^. The pathogenesis of LOS is multifactorial in nature. Venous catheters are thought to be one of the primary causes of LOS. They provide access for the bacteria living on the body’s surface to the bloodstream and make it possible for contaminated intravenous fluids and microorganisms growing on the catheters to cause an infection^13^. To better understand the safety profile and disadvantages of venous catheters in the context of neonatal sepsis we compared the risk factors as well as characteristics of neonates with CRBSI according to the type of venous catheter that was utilized and according to birth weight classification.

Low birth weight has been established as a risk factor for developing sepsis^14^ and very low birth weight infants have been shown by Adatara et al. to be more than twice as likely to develop sepsis in comparison to infants born with a normal weight^15^. Gestational age is besides birth weight the most commonly used parameter to determine an infant’s degree of maturation at birth. Infant maturity is the single most important factor for assessing the risk of an infant to suffer from complications and for that patient to have an unfavorable outcome^16^.

Multiple variables, which we will be exploring in this discussion, make immature infants more susceptible to infections. One of them being physical factors such as the VLBW infant’s skin. It may enhance the microorganism’s ability to grow at the site of the catheter and make this patient group prone to the development of CRBSI. Particularly the decreased thickness of the stratum corneum and the increased insensible water loss results in a moister skin and a more favorable environment for microbial proliferation^17,18^.

Based on the previous research, we suggest that these patients also experience a more complicated hospitalization. This hypothesis can be evaluated by looking at parameters such as the age at termination of parenteral nutrition, duration of antibiotic therapy, and laboratory markers reflecting inflammatory processes.

Infants with below normal birth weight may require prolonged invasive support, which in itself is a risk factor for the development of CRBSI^19^. VLBW infants require IV fluids, medications, and parenteral nutrition more frequently, which in turn exposes them to the risks of venous catheters earlier than their higher birth weight counterparts^20^. Catheters used for the administration of parenteral nutrition are prone to contamination by microorganisms because of the growth-facilitating components in the infusate^21^. For this reason, proper handling of the solution and short infusion times are of great importance. Zingg et al.^22^ has shown treatment and therapy with parenteral nutrition to be linked with an attributable mortality of 11%. Whereas, early introduction of enteral nutrition in VLBW infants is associated with a decreased risk of developing sepsis^23^. These findings are reflected in our results, where the high birth weight CVC group also had the highest age at termination of parenteral nutrition, and the comparatively lower birth weight PVC group had a lower age at termination of parenteral nutrition. This might suggest that parenteral nutrition played a role in the pathogenesis of CRBSI in the CVC group.

The infants receiving a CVC required the longest period of parenteral nutrition and antibiotic therapy. Therefore, we could speculate infants with a CVC to have had a more complicated hospitalization. Based on our findings, we can not exclude that the higher CRP in this study group is related to the gestational maturity, higher birth weight, and their potential ability to mount a more intense immunologic reaction in comparison to the smaller study participants. On the other end of the spectrum, the infants that received a PVC in our study required parenteral nutrition and antibiotic therapy for the shortest time in comparison to infants using other catheter types. This could lead us to believe that the PVC group seemed to have had a less complicated hospitalization, which could be a result of the faster termination of parenteral nutrition, or alternatively could be due to a less severe immunological reaction in this patient group.

Preterm infants suffer from quantitative deficits of immune cells as well as qualitative deficits in the efficiency of killing pathogens^24^. The adaptive immune system in newborns has yet to achieve memory and specificity, which is why newborns rely on their innate immune system and the immunoglobulin Gs that were transferred to the fetus transplacentally from the mother to phagocytose pathogens^25,26^. Additionally, a deficit of proinflammatory cytokines such as interleukin-6 (IL-6) and tumor necrosis factor creates an environment advantageous for infections in preterms^27^. IL-6 in turn stimulates the production of acute-phase proteins. The former has been analyzed in a cohort study with 241 infants by Henrik Doellner et al., who found CRP to be significantly lower in preterm than in term neonates^28^. Similarly, it has been shown that term neonates are three times as likely to have a pronounced CRP response of 60 mg/l than neonates born at 24–27 weeks of gestation^29^. Serial CRP measurements are used to assess the course of an infection, the efficacy of initiated treatment, and the time at which treatment can be terminated^30^. However, since a CRP response by the preterm infants is likely premature, it is unclear whether the low CRP levels are a reliable marker of disease severity.

In our study infants with a higher gestational age also had a significantly higher level of CRP, as can be seen in the infants that received a CVC. As shown in Table 1, infants that had an indwelling PICC were the only ones among the three catheter type groups that had an average birth weight below 1500 g which classifies them as very low birth weight. Despite VLBW being a risk factor for CRBSI, the infants with PICC had the lowest CRP level of any other catheter type group, which is most likely attributed to this group’s immune system immaturity^13^. The prolonged hospitalization time in this group could possibly be explained solely by their physical immaturity.

After stratifying the infants by birth weight of less than or more than 1500 g, laboratory markers reflecting the severity of inflammatory processes like CRP, procalcitonin, and WBC count were not significantly elevated. Therefore, immune system immaturity may not be the only factor influencing elevated CRP in the CVC patient group, but possibly other factors like prolonged invasive care.

The type of microorganism that has been isolated from blood culture was not significantly associated with the type of venous catheter that was used in the therapy and treatment of our infant population. Hence, not one specific type of catheter predisposed to a certain etiological agent causing CRBSI. Also, infants with a birth weight of < 1500 g and > 1500 g did not differ in sepsis etiology.

Since our catheter groups significantly differed in birth weight and gestational age, it is difficult to assess the superiority of any one particular catheter type without physical maturity to act as a confounding factor. As for future directions, we suggest that future studies assess whether there is a correlation between the development of end organ damage consistent with the definition of severe sepsis in patients with CRBSI and the types of vascular catheters that have been used in the care of these patients.

## Conclusions

CRBSI is a serious complication in the NICU, where invasive support is inevitable to provide the best care for newborns. Infants with low birth weight and those who required prolonged parenteral nutrition were most likely to develop CRBSI in our study group. The type of venous catheter was not associated with blood culture results. Also, infants with a birth weight of < 1500 g and > 1500 g did not differ in sepsis etiology. Further research is required to assess venous catheters relative risk of causing sepsis and if the outcome can be traced back specifically to catheter type or patient characteristics.

## Data availability

The data are not publicly available due to ethical and privacy restrictions.
